# Botulinum neurotoxin X lacks potency in mice and in human neurons

**DOI:** 10.1128/mbio.03106-23

**Published:** 2024-02-13

**Authors:** Brieana M. Gregg, Takuhiro Matsumura, Travis G. Wentz, William H. Tepp, Marite Bradshaw, Pål Stenmark, Eric A. Johnson, Yukako Fujinaga, Sabine Pellett

**Affiliations:** 1 Department of Bacteriology, University of Wisconsin, Madison, Wisconsin, USA; 2 Department of Bacteriology, Kanazawa University, Kanazawa, Ishikawa, Japan; 3 Department of Biochemistry and Biophysics, Stockholm University, Stockholm, Sweden; Duke University School of Medicine, USA

**Keywords:** botulinum neurotoxin, BoNT, *Clostridium botulinum*, BoNT/X

## Abstract

Botulinum neurotoxins (BoNTs) are a class of toxins produced by *Clostridium botulinum* (*C. botulinum*) and other species of *Clostridia*. BoNT/X is a putative novel botulinum neurotoxin identified through genome sequencing and capable of SNARE cleavage, but its neurotoxic potential in humans and vertebrates remained unclear. The *C. botulinum* strain producing BoNT/X, Strain 111, encodes both a plasmid-borne *bont/b2* as well as the chromosomal putative *bont/x*. This study utilized *C. botulinum* Strain 111 from Japan as well as recombinantly produced full-length BoNT/X to more fully analyze this putative pathogenic toxin. We confirmed production of full-length, catalytically active native BoNT/X by *C. botulinum* Strain 111, produced as a disulfide-bonded dichain polypeptide similar to other BoNTs. Both the purified native and the recombinant BoNT/X had high enzymatic activity *in vitro* but displayed very low potency in human-induced pluripotent stem cell-derived neuronal cells and in mice. Intraperitoneal injection of up to 50 µg of native BoNT/X in mice did not result in botulism; however, mild local paralysis was observed after injection of 2 μg into the gastrocnemius muscle. We further demonstrate that the lack of toxicity by BoNT/X is due to inefficient neuronal cell association and entry, which can be rescued by replacing the receptor binding domain of BoNT/X with that of BoNT/A. These data demonstrate that BoNT/X is not a potent vertebrate neurotoxin like the classical seven serotypes of BoNTs.

**IMPORTANCE:**

The family of botulinum neurotoxins comprises the most potent toxins known to humankind. New members of this family of protein toxins as well as more distantly related homologs are being identified. The discovery of BoNT/X via bioinformatic screen in 2017 as a putative new BoNT serotype raised concern about its potential as a pathogenic agent with no available countermeasures. This study for the first time assessed both recombinantly produced and native purified BoNT/X for its vertebrate neurotoxicity.

## INTRODUCTION

Botulinum neurotoxins (BoNTs) are a family of the most potent biological toxins known to humans and vertebrates and are produced by anerobic, Gram-positive, spore-forming species of *Clostridia* (1–3). BoNTs are the causative agent of botulism, a potentially lethal paralytic disease, characterized by persistent descending flaccid paralysis (4, 5). The BoNT protein family is genetically diverse, consisting of at least seven distinct antigenic serotypes (BoNT/A-G) with 37%–70% pairwise amino acid identity and over 40 subtypes (6). In terms of function, serotypes and subtypes can differ by neuronal cleavage target, cell entry, potency, and longevity of paralytic effect (6–10).

BoNTs are 150-kDa proteins composed of three major functional domains. After expression as a 150-kDa polypeptide, BoNTs undergo post-translational proteolytic activation via environmental or host proteases into a disulfide-linked ~50-kDa light chain (LC) and ~100-kDa heavy chain (HC) dichain protein (4, 6). The dichain proteins specifically target human and vertebrate neurons. Intoxication occurs primarily at cholinergic nerve terminals, where the HC C-terminal domain (H_C_) binds specific ganglioside and protein receptors on the neuronal cell surface, resulting in endocytosis of the holotoxin (5, 11). The N-terminal HC domain (H_N_) then facilitates translocation of the LC into the cytosol, where the LC is released from the HC by disulfide bond reduction (12) and cleaves SNARE (soluble N-ethylmaleimide-sensitive factor attachment protein receptors) proteins. SNARE proteins are an essential component of the exocytosis machinery, and the specific cleavage by BoNTs disables this function and thereby prevents fusion of acetylcholine-containing vesicles to the membrane of presynaptic nerve terminal (13, 14). Absence of acetylcholine signaling in the synaptic cleft results in the persistent flaccid muscular paralysis characteristic of botulism.

Formal serotype classifications (BoNT/A-G) are based on the absence of cross-neutralization by antisera derived from a given serotype (15), although some variants include chimeric BoNTs or novel BoNTs identified by DNA sequencing only, without toxicity analyses of the entire toxin protein, defying simple classification (16). Increased availability of bacterial genomes through whole genome sequencing has identified non-clostridial BoNT homologs which continue to be discovered. These bioinformatically discovered BoNT homologs and BoNTs share key motifs related to metalloprotease function and a similar multi-domain structure. Briefly, BoNT/Wo (*Weissella oryzae*), Cp1-3 (*Chryseobacterium piperi*), BoNT/Ef (*Enterrococcus faecium*), PMP1 (*Paraclostridium bifermentas*), and PGT1/2 (*Paeniclostridium ghonii*) putative toxin genes were discovered via bioinformatic screens, and their natural target cell types and species are unknown (17–20) . Some BoNT-like toxins have proven SNARE targets outside of vertebrates, such as PMP1 which cleaved mosquito-derived syntaxin and was toxic to mosquito larvae when expressed recombinantly (21).

In contrast to most BoNTs, originally discovered through their association with botulism, the pathogenic potential of bioinformatically discovered BoNT homologs remains unknown without active investigation. While studies have generally been quick to demonstrate the enzymatic activity of the light chain against neuronal SNARE substrates, many unknowns remain regarding the toxicity and activity of the full holotoxin. Such studies are complicated by the regulatory restrictions in place for the production and characterization of native and recombinant BoNTs found in *Clostridium*.

While SNARE cleavage and structural similarities link BoNTs and many BoNT homologs, an important distinguishing factor is the production of the homologs by non-clostridial species, whereas classical BoNTs A-G are expressed by *C. botulinum* species. A unique exception among homologs is BoNT/X, a bioinformatically identified BoNT with unknown toxicity discovered in *C. botulinum* strain, which is genetically more closely related to the non-clostridial homologs than to BoNT/A-G (22). BoNT/X shares ~30% protein sequence identity with other BoNT serotypes but retains conservation at key motifs and the broad domain structure of BoNTs (23). The *bont/x* was discovered in the genome of *C. botulinum* Strain 111 in 2017, which prior to the discovery of *bont/x* was considered a BoNT/B-producing strain isolated from a 1995 case of infant botulism in Japan (24, 25). Antisera raised against BoNT/B was sufficient to ablate toxicity, and analyses of patient samples taken over the course of recovery indicated that the strain could naturally lose the *bont/B2* gene, developing a non-toxigenic phenotype (26). This finding was replicated by a laboratory study wherein serial passage of Strain 111 led to loss of the *bont/B2* containing plasmid and loss of toxicity (27). The entire genome of *C. botulinum* Strain 111 was sequenced in 2015 , and a 2017 study found that while the *bont/b* gene was encoded on a large plasmid, the strain possessed an additional putative *bont* gene cluster on the chromosome, which was named *bont/X* (23). The *bont/x* gene cluster contains NTNH and p47/orfX accessory genes arranged in a unique orientation relative to those generally observed in *bont/a*, *e*, and *f*. These variations further suggest that the *bont/x* gene cluster is significantly distant from the classical *bont* genes (16, 28).

Functional analysis of recombinant BoNT/X LC expressed in *E. coli* revealed that BoNT/X LC is a metalloprotease that cleaves Vesicle-associated Membrane Proteins (VAMP) 1, 2, 3, 4, and 5, as well as the SNARE homolog Ykt6 (23, 29). *In vitro*, the BoNT/X LC displayed ~10-fold higher VAMP1 cleavage efficiency when compared with the BoNT/B LC (29). However, this high potency *in vitro* did not translate to high potency *in vivo*. A recombinant BoNT/X holotoxin was created by sortase ligation of *E.coli*-produced LC-H_N_ and H_C_, and while intramuscular (i.m.) injection of up to 0.5 µg BoNT/X per mouse resulted in local hindlimb paralysis, intraperitoneal (i.p.) injection of 1 µg BoNT/X per mouse resulted in no death or symptoms of botulism (23). These data are in stark contrast to the mouse i.p. lethal dose 50 (LD50) values for BoNT/B1 of 0.21–0.50 ng/kg (30, 31). While sortase ligation is capable of linking two protein domains, this method inherently introduces changes into the protein and should not be used to exclude neurotoxicity of the native putative toxin.

BoNTs and other neurotoxins are defined by their ability to specifically enter neuronal cells and disrupt neurotransmitter release, and while BoNT/X was shown to possess the ability for SNARE cleavage and thus neurotransmitter release disruption, neuronal cell entry has not been demonstrated. Furthermore, the atoxic phenotype of plasmid-cured (PC) *C. botulinum* Strain 111 indicates either a lack of expression or stability of BoNT/X or inability of the toxin to form its active dichain form or inability of BoNT/X LC to reach its target within the neuronal cell. In this study, we sought to further investigate the potential toxicity of BoNT/X. We analyzed full-length BoNT/X produced recombinantly in *E. coli* and *C. botulinum* and examined endogenous production of native BoNT/X by *C. botulinum* Strain 111. Our data indicate that BoNT/X was expressed in Strain 111 as an active dichain, and the toxin displayed high levels of catalytic activity *in vitro* but little to no toxicity to mice *in vivo*. Similarly, recombinant BoNT/X produced in *E. coli* or atoxic *C. botulinum* was able to cleave human VAMPs but displayed inefficient neuronal cell entry into human-induced pluripotent stem cell (hiPSC)-derived neurons. Taken together, these data suggest BoNT/X’s apparent lack of toxicity in mice and human neurons is due to poor or no neuronal cell entry.

## RESULTS

### BoNT/X produced recombinantly in *E. coli* is catalytically active *in vitro* but inefficiently cleaves VAMP2 in neuronal cells

Catalytically inactive BoNT/X_RY_ expressed in *Escherichia coli* (*E. coli*) BL21 (DE3) was used to inoculate and boost mice for production of anti-BoNT/X antiserum. The serum was successfully able to detect the immunogen BoNT/X_RY_ as well as recombinant BoNT/X expressed in atoxic *C. botulinum* strain Hall A hyper tox- (Fig. S1).

**Fig 1:**
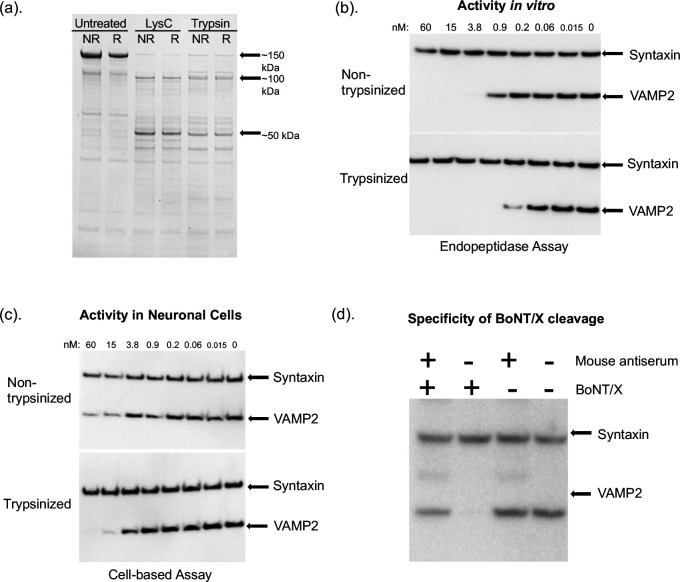
Characterization of recombinant BoNT/X expressed in *E. coli*. (a) Coomassie stained SDS-PAGE gel of BoNT/X expressed in *E. coli* BL21 (DE3), purified via IMAC-Ni^2+^ column, and treated with LysC or trypsin at 250:1 (wt/wt) BoNT/X:LysC or trypsin. R, reduced (with 100 mM DTT); NR, non-reduced. (b) *In vitro* activity of rBoNT/X in hiPSC-derived neuronal cell lysates. Cell lysates were incubated with either trypsinized or non-trypsinized BoNT/X in reducing buffer and were analyzed via western blot for VAMP2 cleavage. Syntaxin was used as a loading control. (c) *In situ* activity of rBoNT/X in hiPSC-derived neurons. Cultured neurons were exposed for 24 hours to serial dilutions of BoNT/X, and cell lysates were analyzed for VAMP2 cleavage via western blot. Estimated concentrations (nM) of BoNT/X are noted. (d) Antibody neutralization cell assay. Trypsin-treated BoNT/X (20 nM) was incubated with anti-BoNT/X antiserum for 1 hour, and hiPSC-derived neurons were exposed to the antibody-BoNT/X mixture, BoNT/X alone, anti-BoNT/X antiserum alone, or culture media alone for 40 hours. Cell lysates were analyzed for VAMP2 cleavage via western blot, using syntaxin as a loading control.

Wild-type (WT) BoNT/X containing a C-terminal his tag (denoted as BoNT/Xwt-his6) was expressed in *E. coli* BL21 (DE3) and purified via the IMAC-Ni^2+^ column. Analysis of the purified rBoNT/X via western blot showed significant aggregation as evidenced by the high-molecular weight smear above the 150-kDa toxin band in the non-reduced sample. Furthermore, toxin treated with LysC or trypsin was detected as two primary bands of ~100 kDa and ~50 kDa, regardless of whether the samples were reduced or not (Fig. 1a), indicating failure of trypsin or LysC to convert the rBoNT/X to a dichain form linked by a disulfide bond. Trypsinized rBoNT/X treated with N-ethylmaleimide to prevent disulfide bond formation did not show significantly different SDS-PAGE band patterns for reduced or non-reduced samples. Therefore, this observed lack of a 150-kDa band in the non-reduced samples may be the result of disulfide bond shuffling as previously proposed (23) or proteolytic degradation by the enzymes meant to activate the toxin.

A previous study found that aggregation of a BoNT/X LC-HC_N_ could be eliminated via mutation to one of the three cysteines in the toxin linker region—C423, C461, or C467—which is in support of the disulfide bind shuffling hypothesis where improper disulfide bonding prevents proper intrachain disulfide bond formation (23). However, point mutations C467A and C461A in full-length BoNT/X_RY_ did not improve aggregation in our un-reduced samples nor did it result in an observable change to the trypsin-cleavage pattern between the mutants and wild-type BoNT/X_RY_ (Fig. S3).

In a hiPSC-derived neuronal cell lysate assay (32), reduced rBoNT/X holotoxin efficiently cleaved VAMP2 and trypsin treatment of the toxin increased cleavage activity by approximately fivefold (estimated EC50 0.8 nM) when compared with non-trypsin-treated samples (estimated EC50 0.16 nM) (Fig. 1b). These data are in agreement with previous data showing the high enzymatic activity of BoNT/X LC (23, 29). Interestingly, this high level of enzymatic activity of rBoNT/X was not observed in cell-based assays using hiPSC-derived neurons, which requires cell entry of the toxin prior to endopeptidase activity (Fig. 1c). Here, serial dilutions of trypsin-treated and non-treated rBoNT/X were only observed to cleave VAMP2 at concentrations greater than 3.8–15 nM, with trypsinized toxin increasing catalytic activity, indicating activation by trypsin treatment. Pre-incubation of trypsin-treated rBoNT/X with polyclonal anti-BoNT/X antiserum before exposure to hiPSC-derived neurons completely abolished VAMP2 cleavage, confirming that the cleavage was specific to BoNT/X activity (Fig. 1d). It has been shown that the BoNT/A LC-H_N_ alone can enter neurons at concentrations greater than 10 nM and cleave SNAP-25 (33); therefore, we cannot exclude the possibility that the observed VAMP2 cleavage by rBoNT/X at high nanomolar concentrations may be due to the non-specific uptake of rBoNT/X or fragments containing the LC rather than targeted translocation into the neuronal cytosol.

Lastly, *E. coli*-derived rBoNT/X was injected i.p. into mice (*n* = 2) at concentrations of ~30 µg/mouse non-trypsin treated and ~15 µg/mouse trypsin treated. Mice were observed for the following 48 hours for symptoms indicative of neuromuscular paralysis. Mice injected with trypsin-treated rBoNT/X displayed no symptoms, although mice injected with non-trypsin-treated samples exhibited mild symptoms but did not die. In a Digit Abduction Score (DAS) Assay, mice (*n* = 2) were injected in the gastrocnemius muscle with ~0.4 µg/mouse trypsin-treated or untreated rBoNT/X and the flaccidity-mediated reduction in digit spread was analyzed (34). There was no detectable paralysis (DAS = 0). Taken together, these data analyzing rBoNT/X produced in *E.coli* indicate little to no neurotoxicity. However, since the rBoNT/X aggregated and was not converted to a disulfide-linked LC-HC dichain, low activity due to protein solubility and stability could not be excluded. The lack of activity after trypsin treatment is likely related to the failure to convert rBoNT/X to a disulfide-linked toxin dichain.

### BoNT/X produced recombinantly in atoxic *C. botulinum* poorly cleaves VAMP2 *in situ* and is atoxic to mice

To produce stable rBoNT/X that undergoes normal post-translational processing, wild-type full-length BoNT/X was expressed in an atoxic *Clostridium botulinum* expression host, strain Hall A hyper tox- (35, 36).

The rBoNT/X was produced as a 150-kDa un-nicked protein in this clostridial strain within 24 hours, with apparent partial degradation ( Fig. S1). Further incubation resulted in loss of the rBoNT/X, indicating that BoNT/X is unstable in culture supernatants of proteolytic *C. botulinum* and that the proteasome of *C. botulinum* strain Hall A hyper tox- does not convert BoNT/X to its dichain form. A small percentage of soluble BoNT/X was recovered in the extract supernatant post-sonication while the majority of the toxin remained associated with the cell pellet. A portion of the extract supernatant containing soluble BoNT/X was kept for further analysis, while the remaining supernatant underwent partial purification via SE (S-300) and IEX (DEAE) chromatography.

**Fig 2:**
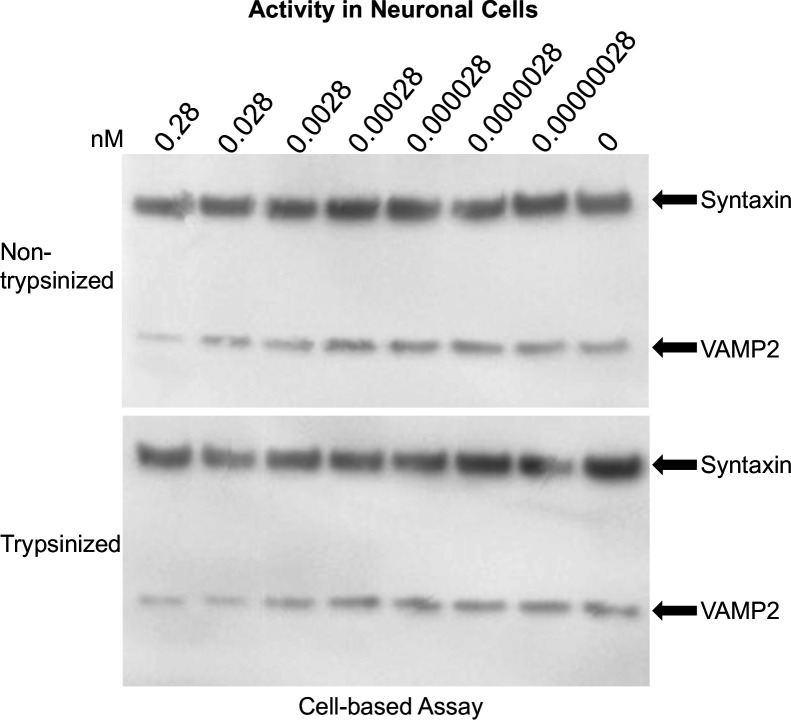
Characterization of recombinant BoNT/X expressed in *C. botulinum. In situ* activity of rBoNT/X extract in hiPSC-derived neurons. Cultured neurons were exposed for 72 hours to serial dilutions of rBoNT/X, and neuronal cell lysates were analyzed for VAMP2 cleavage via western blot. Estimated concentrations (nM) of BoNT/X are noted.

Serial dilutions of trypsin-treated or untreated rBoNT/X extract supernatant (Fig. 2) and partial purification pool (data not shown) were exposed to hiPSC-derived neurons for 72 hours, and cell lysates were examined for VAMP2 cleavage as described previously (32). Only a mild partial decrease of the VAMP2 band was observed, regardless of trypsin treatment, indicating little VAMP2 cleavage. These data indicate that, similar to *E.coli*-produced rBoNT/X, rBoNT/X produced in an endogenous expression host also has little to no neurotoxic activity. However, even after production in an endogenous expression host, there was a lack of normal post-translational processing to the dichain form, and partial degradation of the toxin was apparent. Mice (*n* = 4) were injected with 500 µL of trypsin-treated or non-treated crude BoNT/X extract or partially purified BoNT/X DEAE pool. Based on densitometry, the estimated injection concentration for the non-treated rBoNT/X extract and rBoNT/X DEAE pool was 2.5 µg/mouse. Mice injected with BoNT/X extract displayed initial mild distress, and mice injected with partially purified BoNT/X from DEAE chromatography showed moderate signs of distress (labored breathing, lethargy) immediately after injection, but both groups quickly recovered and displayed no further symptoms of botulinal intoxication throughout the remaining observation period. The quick onset of initial symptoms seen in both groups is not consistent with botulinal intoxication.

### *C. botulinum* Strain 111 endogenously expresses BoNT/X at similar levels to BoNT/B and proteolytically processes native BoNT/X to its dichain form

**Fig 3:**
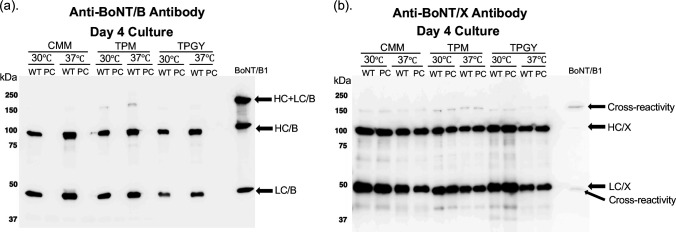
Production of BoNT/X and BoNT/B by WT and PC *C. botulinum* Strain 111. Cultures were grown anaerobically at 30°C or 37°C in 5 mL of cooked meat medium (CMM), tryptone peptone glucose yeast extract (TPGY), or toxin production media (TPM) for 4 days. Reduced (via 100 mM DTT) samples were analyzed via SDS-PAGE with anti-BoNT/B antibody (a) or anti-BoNT/X antiserum (b). Fifty nanograms of BoNT/B1 toxin was used as a positive control for both. Where noted, the BoNT/B holotoxin and LC weakly cross-reacted with the anti-BoNT/X antiserum. HC, heavy chain; LC, light chain; HC+LC, holotoxin.

**Fig 4:**
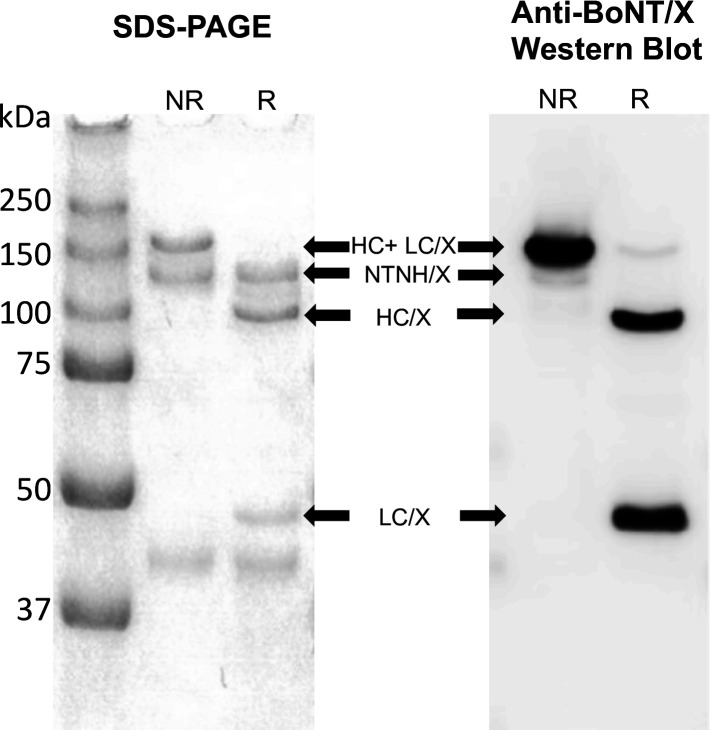
BoNT/X purified from *C. botulinum* Strain 111. BoNT/X was purified from plasmid-cured Strain 111 cultured 4 days in TPGY. Reduced (R) and non-reduced (NR) samples were analyzed via SDS-PAGE gel (left) or SDS-PAGE probed with mouse anti-BoNT/X antiserum (right). HC+LC, holotoxin; HC, heavy chain; LC, light chain.

Native BoNT/X was analyzed to definitively determine whether BoNT/X may have human and vertebrate neurotoxicity. The native *C. botulinum* Strain 111 is a bivalent toxin strain isolated from a clinical botulism case in Japan, which encodes both a chromosomal *bont/*x and a plasmid-borne *bont/b2* (23). Strain 111 has previously been shown to readily lose the *bont/b2*-containing plasmid via repeated culture passaging (27); therefore, to analyze endogenous BoNT/X expression, *C. botulinum* Strain 111 was serially passaged until the *bont/b2*-bearing plasmid was lost. Plasmid loss was confirmed via PCR using primers recognizing the *bont/b2* gene and the *bont/x* genes (data not shown) and by western blots probing with anti-BoNT/B antibody (Fig. 3). Western blot probing with anti-BoNT/X antiserum showed that BoNT/X is endogenously expressed in *C. botulinum* Strain 111 at levels consistent with other BoNTs and similar to expression of BoNT/B2 in wild-type Strain 111 (Fig. 3). There was no apparent change in quantity of BoNT/X between the WT and PC samples under a variety of tested growth conditions and media, suggesting no loss in toxin expression after the plasmid-curing process. Furthermore, native BoNT/X was fully converted to its dichain form, as indicated by the 100-kDa HC and 50-kDa LC bands in the reduced samples on the western blot (Fig. 4).

### Culture supernatant of plasmid-cured Strain 111 containing BoNT/X is non-toxic to mice

Culture supernatants of WT and PC samples of Strain 111 grown for 4 days in either CMM, TPM, or TPGY at 30°C or 37°C were injected i.p. into mice (*n* = 2). Mice were observed for death or other motor impairment symptoms consistent with botulism over the following 7-day period. The majority of mice injected with WT Strain 111 died within 3 hours post-injection, and all died within 24 hours (Table 1). There were no deaths nor symptoms of botulism in any of the mice injected with PC Strain 111 (Table 1). As WT Strain 111 was shown to endogenously express BoNT/B and BoNT/X at similar quantities (Fig. 3), the lack of toxicity of PC samples in the mouse bioassay (MBA) is further indication that BoNT/X is not a potent vertebrate neurotoxin.

**TABLE 1:** MBA of 4-day culture supernatants of WT or PC *C. botulinum* Strain 111

Media	Day 1 (alive/tested)	Day 7 (alive/tested)
CMM 30°C WT	0/2	0/2
CMM 30°C PC	2/2	2/2
CMM 37°C WT	0/2	0/2
CMM 37°C PC	2/2	2/2
TPM 30°C WT	0/2	0/2
TPM 30°C PC	2/2	2/2
TPM 37°C WT	0/2	0/2
TPM 37°C PC	2/2	2/2
TPGY 30°C WT	0/2	0/2
TPGY 30°C PC	2/2	2/2
TPGY 37°C WT	0/2	0/2
TPGY 37°C PC	2/2	2/2

### BoNT/X is post-translationally processed to a dichain form in *C. botulinum* Strain 111

BoNT/X was purified from *C. botulinum* Strain 111 as the minimal progenitor toxin complex (M-PTC, final concentration of 340 μg/mL), as attempts to dissociate BoNT/X from its non-toxic non-hemagglutinin. (NTNH) protein by ion exchange chromatography under alkaline conditions resulted in BoNT/X being degraded ( Fig.S4). SDS-PAGE showed that the isolated BoNT/X M-PTC in the absence of DTT forms two distinct bands at ~140 kDa and ~150 kDa (Fig. 4, left). The former is likely NTNH/X, as treatment with DTT resulted in no change in band size, whereas the ~150-kDa band separated into distinct 100-kDa and 50-kDa bands upon reduction with DTT, indicating the presence of the native BoNT/X as a LC-HC dichain in culture supernatant. The identity of the 150-kDa and 100- and 50-kDa bands was further confirmed by western blot (Fig. 4 right).

These data represent the first confirmation that BoNT/X is expressed in its native strain and that it is post-translationally processed by host strain proteases to its fully active dichain form.

### Endogenously produced BoNT/X displays high catalytic activity

**Fig 5:**
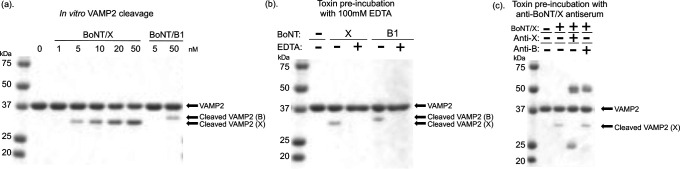
*In vitro* activity of native BoNT/X. (a) Western blot showing *in vitro* GST-human VAMP2 (2-94) cleavage by reduced BoNT/X and BoNT/B1 (Okra). The toxins were exposed to VAMP2 for 45 minutes before analysis. (b) Western blot of *in vitro* cleavage of GST-human VAMP2 with 5 nM reduced BoNT/X or 50 nM reduced BoNT/B either pre-incubated with 100 mM EDTA for 30 minutes (+) or are left untreated (−). (c) Western blot showing inhibition of GST-human VAMP2 cleavage by 5 nM reduced BoNT/X if pre-incubated for 30 minutes with 1 μg anti-BoNT/X antiserum (+Anti-X) but not with 1 μg anti-BoNT/B rabbit polyclonal antibody (+Anti-B).

Dilutions of reduced BoNT/X ranging from 0 to 50 nM alongside dilutions of reduced BoNT/B1 (Okra) were exposed to the extracellular domain of GST human VAMP2 in an endopeptidase assay (Fig. 5a) (37). Analysis of the VAMP2 substrate via western blot confirmed that, as with recombinantly expressed BoNT/X, endogenous BoNT/X is highly catalytically active and displays ~10-fold higher cleavage than BoNT/B1. To further confirm that the observed VAMP2 cleavage is due to catalytic activity of the BoNT/X LC, BoNT/X was treated with the chelating agent ethylenediaminetetraacetic (EDTA) before repeating the VAMP2 endopeptidase assay.

This resulted in a lack of cleavage in pre-treated BoNT/X samples (Fig. 5b). Additionally, preincubation of 5 nM BoNT/X with either mouse anti-BoNT/X antiserum or anti-BoNT/B antibodies demonstrated that BoNT/X cleavage of VAMP2 is inhibited by anti-BoNT/X antiserum but unaffected by anti-BoNT/B antibodies (Fig. 5c). This suggests BoNT/X is an active metalloprotease whose activity can be suppressed through the addition of a chelating agent or anti-BoNT/X antiserum.

### BoNT/X does not bind the PC12 cellular membrane

**Fig 6:**
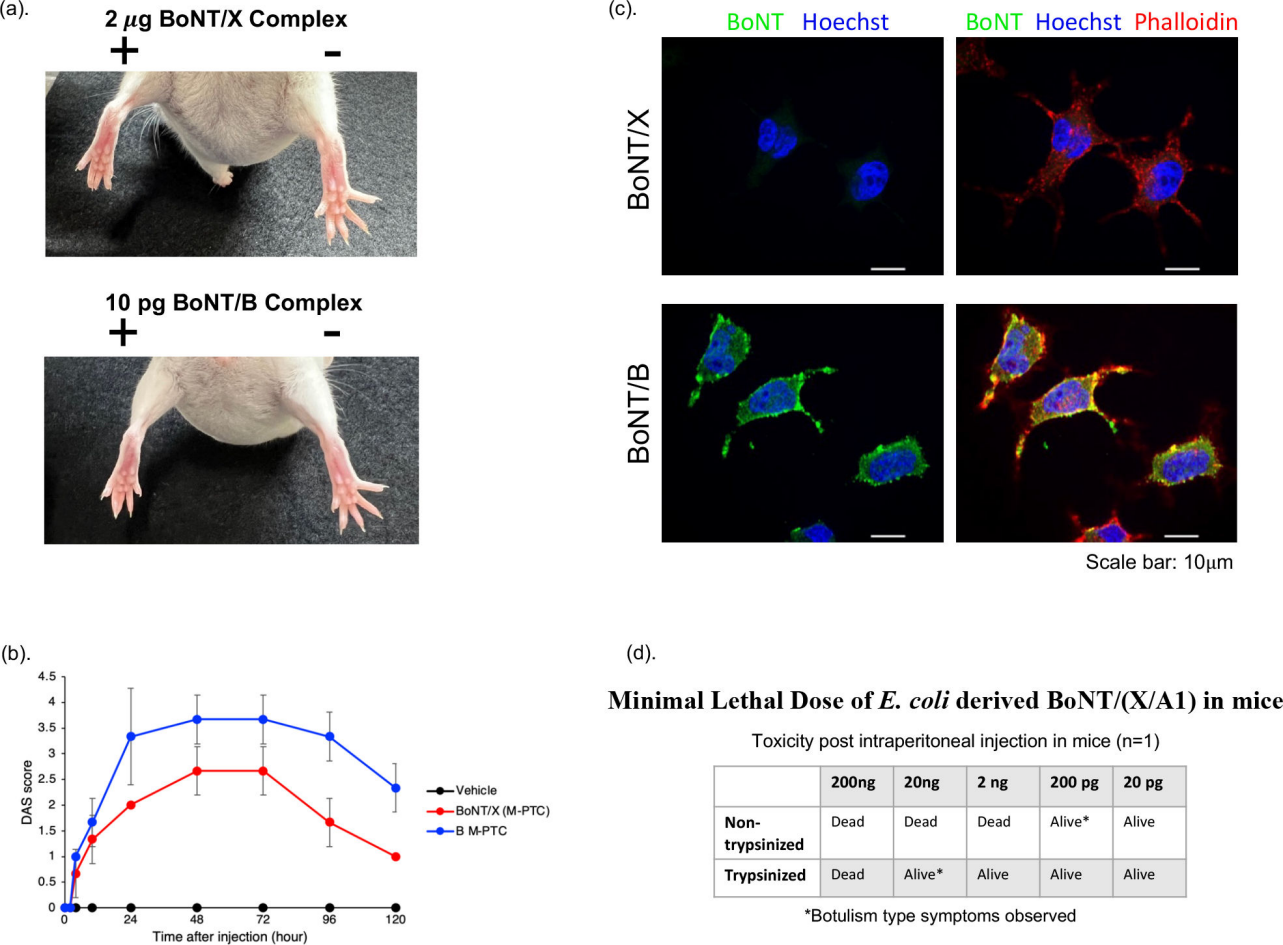
Native BoNT/X causes mild local paralytic effects on mice. (a) Mice (*n* = 3) were injected into the left gastrocnemius muscle with either 2.0 μg BoNT/X complex or 10 pg BoNT/B (Okra). The right hindlimb was injected with vehicle. Shown are representative images of digital abduction at 24 hours post-injection, where both BoNT/X and BoNT/B complexes result in moderate muscle paralysis. (b) Reduction in reflexive spread of digits was quantified via the DAS, with average values and standard deviations depicted in the graph (*n* = 3). (c) Confocal microscopy depicting the lack of native BoNT/X binding to neuronal PC12 (rat) cells. (d) Minimal lethal dose (MLD) of recombinant X(LC-HC_N_)/A(HC_C_) chimera, expressed in *E. coli*. Estimated concentration BoNT/(X/A1) holotoxin is indicated based on densitometry of total purified protein.

Rat pheochromocytoma PC12 cells were exposed to 300 nM BoNT/X or BoNT/B for 1 hour before fluorescent imaging in order to assess the ability of BoNT/X to bind the neuronal cell membrane (Fig. 6c). In addition to fluorescent antibodies targeting BoNT/X and BoNT/B (green), antibodies binding DNA (Hoechst, blue) and F-actin (Phalloidin, red) were used. There was no observed binding of BoNT/X to the cell membrane.

### BoNT/X M-PTC exhibits low potency in mice

*In vivo* toxicity of the native BoNT/X M-PTC was tested via MBA. Mice (*n* = 3) were injected i.p. with an estimated 5 µg/mouse or 50 µg/mouse BoNT/X M-PTC and monitored for symptoms indicative of botulism or death during the following 4 days (Table 2). No mice displayed symptoms nor did any die after injection. In a DAS assay (*n* = 3 mice), the left hindlimb was injected i.m. with 2 µg of BoNT/X, and the right hindlimb was injected with an equivalent volume of vehicle alone. In control mice, 10 pg of BoNT/B (Okra) was similarly injected alongside the vehicle. As shown in Fig. 6a, mice injected with 2 µg BoNT/X and 10 pg BoNT/B both displayed a reduction in reflexive digit spread 24 hours post-injection, although the average DAS score for mice injected with BoNT/X remained lower than BoNT/B over the course of 5 days (Fig. 6b).

**TABLE 2:** Mouse Bioassay of native BoNT/X isolated from plasmid-cured Strain 111

BoNT/X	Day 1 (alive/tested)	Day 4 (alive/tested)
5 µg/mouse	3/3	3/3
50 µg/mouse	3/3	3/3

### Chimeric BoNT/(X/A1)

To further explore BoNT/X non-toxicity, a recombinant X(LC-HC_N_)/A(HC_C_) [denoted as BoNT/(X/A1)] chimera was expressed in *E. coli*, affinity purified, and trypsinized/non-trypsinized. BoNT/(X/A1) ( Fig. S5) was serially diluted from 200 ng to 20 pg and injected into mice to determine a MLD (Fig. 6d) . MLDs were 200 ng for the trypsinized and 2 ng for the non-trypsinized BoNT/(X/A1), indicating the X LC-HC_N_ is toxic to mice upon proper delivery to the motor neuron. Similar to *E. coli*-produced BoNT/X holotoxin, trypsin treatment of BoNT/(X/A1) resulted in 100-kDa and 50-kDa bands on SDS-PAGE gels under both reduced and non-reduced conditions, indicating unlinked dichain formation (Fig. S5). The data confirm that the trypsin-treated, *E. coli*-produced rBoNT/X fails to result in a disulfide-linked dichain. Overall, these results are consistent with a previous report (23) showing greater activity of a sortase-linked BoNT/X(LC-HC_N_)/A(HC_C_) chimera in cultured neurons and further indicate that replacement of the HC_C_ domain of BoNT/X with HC_C_/A1 increases toxicity.

## DISCUSSION

The discovery of BoNT/X via bioinformatic screen in 2017 as a proposed new BoNT serotype raised concerns about its potential as a pathogenic agent with no available countermeasures (23). Initial experiments suggested that recombinant BoNT/X LC displayed higher cleavage efficiency than BoNT/B *in vitro* (23, 29). In addition, sortase-ligated BoNT/X holotoxin exhibited some toxicity in mice, causing mild local paralysis after injection of 0.5 µg into the gastrocnemius muscle, but no systemic symptoms after intraperitoneal injection (23, 29). These data, however, could not exclude the potential toxicity of native BoNT/X, since recombinantly expressed and enzymatically ligated BoNT/X may differ in toxicity from the native toxin due to potential insufficient processing of the toxin within the recombinant expression system or as a consequence of conformational changes to BoNT/X following the sortase linking reaction. In addition to these data, no information was available on whether the native *C. botulinum* Strain 111 produces full-length, stable BoNT/X in detectable quantities. As a result of this potential toxicity, BoNT/X was classified as a Tier 1 Select Agent alongside BoNT serotypes A-G.

This study for the first time assessed both recombinantly produced and native purified BoNT/X for its potential as a vertebrate neurotoxin. Purified native and rBoNT/X were tested for enzymatic activity *in vitro*, for cell entry and enzymatic function inside cells in neuronal cells, and for full biologic activity in mice. Our results further provide the first confirmation that BoNT/X is expressed by *C. botulinum* Strain 111 at levels comparable to BoNT/B (Fig. 3). Importantly, while disulfide-bonded dichain formation was not observed for rBoNT/X either by our group (Fig. S1) or by others (23, 38), native BoNT/X was proteolytically processed into a ~150-kDa active dichain in Strain 111, as evidenced by reduction of the 150-kDa toxin to the 100-kDa and 50 kDa HC and LC (Fig. 4).

*C. botulinum* Strain 111 is a dual-toxin-producing strain, with the *bont/x* localized on the chromosome and a *bont/b2* localized on a plasmid. Previous analysis of this strain revealed an atoxic phenotype upon plasmid curing (26, 27), which led to the conclusion that *bont/x* is either silent or BoNT/X is non-toxic. Analysis of the production of native BoNT/X in either the *C. botulinum* Strain 111 wild type or the plasmid cured strain unambiguously showed strong expression of BoNT/X (Fig. 3). These data suggest that native BoNT/X, as produced by its native strain, is not toxic to vertebrates or humans.

In an *in vitro* endopeptidase assay, native BoNT/X holotoxin cleaved VAMP2 more efficiently than BoNT/B at the same concentrations (Fig. 5a), confirming previous studies demonstrating the high catalytic activity of rBoNT/X LC (23, 29, 38). Furthermore, the SNARE cleavage after exposure to BoNT/X holotoxin could be halted either by preincubation with anti-BoNT/X antiserum (Fig. 1d and 5c) or EDTA (Fig. 5b), indicating the observed cleavage is specific to the metalloproteolytic activity of BoNT/X LC. Similarly, full-length rBoNT/X resulted in sensitive VAMP2 cleavage, which was still observable with estimated EC50 values of ~0.8 nM and ~0.15 nM for non-trypsin-treated and trypsin-treated samples, respectively (Fig. 1b, EC50 values quantitated from duplicate assays). This compares to EC50 values of ~2 nM for BoNT/F1 and ~16 nM for BoNT/B1 in the same assay.

With confirmation that BoNT/X is expressed endogenously in *C. botulinum* Strain 111 and is both capable and highly efficient at cleaving mammalian neuronal SNARES *in vitro*, the question of the cause of the apparent lack of *in vivo* toxicity of plasmid cured Strain 111 remains. Both purified native and rBoNT/X were examined for their ability to cause SNARE cleavage inside cultured neurons. In hiPSC-derived neuronal cells, rBoNT/X either did not cleave VAMP2 (Fig. 2), or only partially cleaved VAMP2 at high nM concentrations (Fig. 1c). Since at nanomolar concentrations, non-specific entry and cleavage by LC or LC-containing fragments of the BoNT cannot be ruled out, these data indicate low or no activity of rBoNT/X in cultured human neurons, which have previously been shown to be exquisitely sensitive to BoNT/A, capable of detecting fM amounts (32). One possible explanation for the lack of neuronal toxicity may be BoNT/X holotoxin’s inability to efficiently bind and initiate translocation of the LC into neuronal cytosol. An initial step of BoNT intoxication involves binding of the toxin HC_C_ to gangliosides and protein receptors on the surface of the neuron (39, 40). The classical serotypes BoNT/A-G exhibit varying binding affinities to the most common mammalian neuronal gangliosides GT1b, GM1a, GD1a, and GD1b (41, 42). However, a preprint manuscript by Martínez-Carranza et al. found that rBoNT/X holotoxin displayed very weak binding against these four gangliosides *in vitro* (38). In our own study, endogenously expressed BoNT/X failed to bind neuronal PC12 cells (Fig. 6c), whereas BoNT/B sequestered to the neuronal cell membrane as expected.

Investigations of potential botulism-like toxicity of recombinant and native BoNT further indicated the absence of any systemic toxicity and only very mild local paralysis at very high concentration. Zhang et al. (23) previously reported that i.m. injection of 0.5 µg/mouse of sortase-linked rBoNT/X LC-H_N_ induced local paralysis in mice. Interestingly, we did not observe any detectable hindlimb paralysis (DAS = 0) following i.m. injection of ~0.4 µg/mouse rBoNT/X holotoxin isolated from *E. coli*. The low activity or lack of local paralysis may be in part due to the aggregation and instability of rBoNT/X, which was present throughout the purification process, and failure to form the more active disulfide-bonded dichain (Fig. 1a). However, 2 µg of native BoNT/X , which purified as a disulfide-bonded dichain molecule (Fig. 4), did produce mild paralysis in mice (average DAS = 2 at 24 hours post-injection) (Fig. 6a and b). This paralysis compares to a stronger paralysis caused by injection of 10 pg/mouse of BoNT/B1, suggesting that native BoNT/X requires over 200,000-fold more toxin to produce local paralysis *in vivo* than BoNT/B. Interestingly, mice injected intraperitoneally with up to ~50 µg native BoNT/X, ~30 µg rBoNT/X (expressed in *E. coli)*, or ~2.5 µg rBoNT/X (expressed in *C. botulinum* Hall A-hyper tox-) did not result in any mouse deaths. This indicates that native BoNT/X is at least 10 million-fold less toxic than BoNT/A in mice when injected intraperitoneally. The lack of neuronal cell association in the PC12 cell model (Fig. 6c) and the high enzymatic activity of the LC indicate that the lack of toxicity is due to a failure of BoNT/X to enter human or vertebrate neurons. In fact, replacement of the HCc domain of rBoNT/X with the HCc domain of BoNT/A1 resulted in systemic toxicity in mice (Fig. 6d), demonstrating that the translocation domain and LC of BoNT/X are functional neurotoxin domains, while the receptor binding domain fails to specifically recognize and bind to neuronal cells.

It is interesting to speculate that BoNT/X’s apparent lack of toxicity to mammalian neurons is the result of the toxin targeting another cell type or perhaps another class of vertebrate or non-vertebrate organisms. Although BoNT/X is expressed by a *Clostridium* species and was therefore proposed to be a novel BoNT, it genetically groups more closely with the newly emerging class of non-clostridial BoNT homologs, many of which currently have undefined target species (23). PMP1 is one exception in this lineage, as the recombinant toxin has been shown to cleave neuronal SNARES in *Anopheles* mosquito larvae (21). As BoNT/X shares more protein sequence identity with PMP1 than the seven classic BoNT serotypes, it is possible that identifying BoNT/X’s target cell type may require looking outside of vertebrate neurons. Considering that *C. botulinum* Strain 111 is producing a second, functional BoNT/B toxin, it is also possible that *bont/x* is a defunct gene in this strain that is maintained due to its incorporation into the chromosome and lack of direct targeting of *bont*-gene clusters by endogenous CRISPR systems of *C. botulinum* (43).

This study sought to clarify whether the proposed novel BoNT/X exhibits toxicity comparable to other BoNTs and poses a threat to human health and safety. Whether expressed in the native *C. botulinum* Strain 111 or recombinantly in *C. botulinum* or *E. coli*, our data show that BoNT/X is not a potent vertebrate neurotoxin. Furthermore, these data indicate that the lack of toxicity by rBoNT/X is due to a combination of failure of neuronal cell binding and a failure to form a disulfide-bonded dichain. However, the native BoNT/X was converted to a disulfide-bonded dichain and remained unable to cause systemic botulism in mice at 50 µg, suggesting that the lack of toxicity of native BoNT/X is primarily due to the lack of neuronal cell binding and entry. Determining preferred receptor targets for BoNT/X binding will be an important next step in further understanding the utility of these toxins to the bacteria expressing them and may inform potential uses of BoNT/X for pharmaceutical or research applications.

## MATERIALS AND METHODS

Our Wisconsin laboratory and personnel are registered with the CDC Federal Select Agent Program for research involving botulinum neurotoxins and botulinum neurotoxin-producing strains of clostridia. The research program, procedures, occupational health plan, documentation, security, and facilities are closely monitored by the University of Wisconsin—Madison Biosecurity Task Force, University of Wisconsin—Madison Office of Biological Safety, the University of Wisconsin—Madison DURC subcommittee, and at regular intervals by the CDC and the Animal and Plant Health Inspection Service (APHIS) as part of the University of Wisconsin—Madison Select Agent Program. All personnel have undergone suitability assessments and completed rigorous and continuing biosafety training, including biosafety level 3 (BSL3) and select agent practices before participating in laboratory studies involving botulinum neurotoxins and neurotoxigenic *C. botulinum* strains. All recombinant DNA protocols for the construction of the recombinant BoNT genes and their expression in *C. botulinum* strains have been approved by the University of Wisconsin Institutional Biosafety Committee (IBC), and specific experiments were approved by the Federal Select Agent Program. A dual-use research of concern (DURC) risk mitigation plan has been established and approved by the University of Wisconsin—Madison DURC Subcommittee and NIAID for these experiments. Animal studies involving BoNT select agents were approved by the University of Wisconsin—Madison IACUC.

Our laboratory in Kanazawa University has the permission to possess and handle *Clostridium botulinum* and botulinum neurotoxins (Class II pathogens) by the Minister of Health, Labor and Welfare of Japan, pursuant to the provisions of the Cabinet Order of Japan. The research program and engaged persons are also authorized and closely monitored by the Kanazawa University Safety Committee for pathogen. Animal studies were conducted under the applicable laws and guidelines for the care and use of laboratory animals at the Kanazawa University. They were approved by the Animal Experiment Committee of the Kanazawa University (AP-214252).

### Bacterial strains, media, and reagents

Culture media reagents were purchased from Sigma-Aldrich (St. Louis, MO), BD Difco (Franklin Lakes, NJ), and Fujifilm (Osaka, Japan). Unless otherwise noted, antibodies for western blot analysis were produced in the lab (in the case of mouse-anti BoNT/X and rabbit-anti BoNT/B polyclonal antibodies) or purchased from Santa Cruz Biotechnology Inc. (Dallas, TX) and Jackson ImmunoResearch (West Grove, PA). Antibiotics (chloramphenicol, cycloserine, kanamycin, and thiamphenicol) were purchased from Sigma-Aldrich and used at the following concentrations: in *E. coli*, kanamycin 50 µg/mL, chloramphenicol 25 µg/mL in agar, and 12.5 µg/mL in liquid media and *C. botulinum*, cycloserine 250 µg/mL and thiamphenicol 15 µg/mL.

*E. coli* was grown at 37°C in either liquid LB agitated at 225 rpm or on LB agar plates supplemented with corresponding antibiotics for plasmid selection. *E. coli* 10β and BL21 (DE3) were purchased from New England Biolabs (Ipswich, MA), and the donor strain *E. coli* CA434 was kindly provided by N. Minton (University of Nottingham, UK).

*C. botulinum* cultures were grown in static nitrogen-flushed hungate tubes, and handling of culture was carried out within an anaerobic chamber (Forma Anerobic System, Marietta, OH; atmosphere consisted of 80% N_2_, 10% CO_2_, and 10% H_2_). Larger quantities of culture for purification were grown statically in aerobic conditions. Cultures were grown in the indicated liquid media or agar plate formulated as follows: TPGY)—5% Trypticase peptone, 0.5% Bacto peptone, 0.4% glucose, 2% yeast extract, and 0.1% L-cysteine HCl (pH 7.3–7.4); TPM—2% Trypticase peptone, 0.5% glucose, and 1% yeast extract (pH 7.3); peptone yeast glucose media (PYG)—2% proteose peptone, 0.5% yeast extract, 0.5% glucose, and 0.025% sodium thioglycolate, pH 7.0; CMM—0.1 g/mL H_2_O supplemented with 0.3% glucose and 0.2% soluble starch.

*C. botulinum* Hall A hyper tox- (35) is an atoxic, non-sporulating *C. botulinum* expression host. Using Clostron (36), an intron was introduced into the A1 toxin gene and confirmed via PFGE and southern blotting. Western blotting and mouse bioassay further confirmed the lack of toxin production.

### Production of BoNT/X-specific polyclonal antibody

Catalytically inactive BoNT/X (BoNT/X_RY_) was produced in *E. coli* BL21 (DE3) essentially as described previously (23). *E. coli* cultures were grown in a shaker flask instead of a bioreactor, and after induction, cells were incubated for 19 hours and lysed using a French press. Culture lysate was clarified by centrifugation and passed through a 5-mL HisPur Ni-NTA agarose (Thermo, Waltham MA, #88222) column to produce purified BoNT/X_RY_. Four female ICR mice were injected with 20 µg/mouse of the catalytically inactive holotoxin in 0.4 mL of alhydrogel intraperitoneal and 0.1 mL subcutaneous and boosted in the same manner after 2 weeks. Seven days post-boost, the mice were bled via the maxillary vein and serum was prepared. Specificity of the mouse serum was tested by western blot and was successfully able to detect recombinant BoNT/X expressed in *C. botulinum* Hall A hyper tox-.

### Plasmid curing of *C. botulinum* Strain 111

Glycerol stock of *C. botulinum* Strain 111 (Osaka Prefecture University) was anaerobically cultured in 10 mL (PYG) media for 3 days at 37°C. Whole culture was passaged into fresh media every 3 days to cure the strain of the *bont/b2*-containing plasmid. For verification of plasmid loss, serially passaged culture was spread onto Brucella HK plates and grown anaerobically at 37°C for 24 hours. Single colonies were then grown anaerobically in 5 mL CMM at 37°C. After day 3, aliquots of cultures were centrifuged 5,000 *g* for 10 minutes at room temperature and serotype-specific (BoNT/B) PCR was performed to confirm the loss of the plasmid (data not shown). Western blots further confirmed the loss of the *bont/b2*-containing plasmid from Strain 111. One sample each of the WT and PC colonies verified by serotype-specific PCR were grown up to 4 days in CMM, TPM, or TPGY liquid media. Samples were grown at either 30°C or 37°C, and whole culture aliquots of each were taken at days 1 and 4. The anti-BoNT/B western blot primary antibody was anti-BONT/B1 Rb/PC, and the secondary antibody was anti-rabbit IgG HRP. The anti-BoNT/X western blot primary antibody was BoNT/X-specific polyclonal antiserum produced in mice, and the secondary antibody was anti-mouse IgG HRP. BoNT/B1 toxin control in both western blots was 50 ng BoNT/B1.

### Expression and purification of rBoNT/X

#### *E. coli* BL21 (DE3)

The gene for catalytically inactive BoNT/X was encoded on the *E. coli* expression plasmid pET22b. This gene was altered via site-directed mutagenesis to the catalytically active form by converting amino acids A360R and F363Y. This resulted in wild-type BoNT/X which was his-tagged on the C-terminus (denoted as BoNT/Xwt-his_6_) and expressed in *E. coli* BL21 (DE3). Cells were lysed by sonication on ice in 80 mL of 50 mm Hepes buffer pH 7.2 containing 500 mM NaCl, 45 mM imidazole, 20 mg lysozyme, protease and phosphatase inhibitor (Pierce A32962), and deoxyribonuclease I (Sigma-Aldrich, D5025). The concentrated extract was passed through a 5-mL HisPur Ni-NTA agarose (Thermo 88222) column, and bound BoNT/X was eluted with sonication buffer containing 250 mM imidazole. Purified BoNT/X was treated with trypsin at ~350:1 (wt:wt) toxin:trypsin or toxin:lysC for 15 minutes at room temperature. Non- trypsin-treated and trypsin-treated BoNT/X samples were then reduced via DTT or not reduced before analysis by SDS-PAGE and western Blotting.

#### *C. botulinum* hall A hyper tox-

The gene for catalytically active BoNT/X was cloned into a pMTL8000 series shuttle vector pMTL83152 (44), transformed into the *E. coli* conjugative donor strain CA434, and conjugated into *C. botulinum* Hall A hyper tox- as previously described (45, 46). After mating, cells were resuspended and plated onto fresh media supplemented with 15 µg/ml thiamphenicol (selection for vector plasmid) and 250 µg/mL cycloserine (selection for *C. botulinum*). Transconjugant colonies were selected at random and screened for the presence of the expression vector via plasmid isolation and restriction digest, and for toxin production via western blot.

A 6-L static culture of Hall A-hyper/tox^−^ (rBoNT/X) was grown 24 hours at 37°C in liquid TPGY supplemented with 15 µg/mL thiamphenicol. Cells from the 24-hour culture were collected by centrifugation (7,500 *g*, 10°C, 5 minutes) and stored at −80°C for further processing. Cell pellets were removed from −80°C, thawed, and evenly suspended in 40 mL of sonication/extraction buffer [200 mM sodium/potassium phosphate buffer pH 6.0 containing 10% glycerol, 20 mM DTT, 1.5 mg RNAse A, 2 mg DNAse, and protease inhibitor cocktail (Pierce #A32922)]. The suspension was sonicated for 3 minutes in total on time (10 sec on/30 sec off) at 70% amplitude on ice. For SE chromatography, the suspension was centrifuged at 15,000 *g* for 20 minutes at 10°C. The supernatant was sequentially filtered through 0.45-micron and 0.22-micron filters and the filtered extract applied to a Sephacryl S-300 (Cytiva, Marlborough, MA) column 2.5 × 91 cm equilibrated with 25 mM sodium citrate buffer pH 5.5 containing 10 mM DTT. The column was washed overnight with pH 5.5 citrate buffer containing DTT at room temperature. The optical density of fractions was measured, and peak fractions were analyzed by SDS-PAGE and western blot. Fractions containing BoNT/X were pooled and precipitated with ammonium sulfate (39 g/100 mL). For DEAE chromatography pH 5.5, the precipitate from the S-300 pool was collected by centrifugation (12,000 *g*, 4°C, 20 minutes) and the pellet dissolved in 4 mL of 50 mM sodium citrate buffer pH 5.5 containing 50 mM DTT. The solution was centrifuged at 12,000 *g*, 20°C, for 15 minutes, and the clarified supernatant was applied to a DEAE sephadex A50 column 2.5 × 18 cm equilibrated with 50 mM sodium citrate pH 5.5 + 50 mM DTT. Fractions were analyzed by SDS-PAGE and western blot for the presence of BoNT/X. Fractions from the first peak containing the majority of BoNT/X were pooled and precipitated with ammonium sulfate (39 g/100 mL and stored at 4°C).

### Expression and purification of native BoNT/X

*C. botulinum* Strain 111 cured of the plasmid containing the *bont/b2* gene was inoculated into liquid TPGY media and grown statically at 30°C for 4 days. The culture supernatant was ammonium sulfate (60%) precipitated and stored overnight at 4°C. Precipitate was collected by centrifugation (10,000 *g*, 4°C, 20 minutes), and the pellet was solubilized in 50 mM sodium phosphate buffer pH 6.0. RNAse A (Sigma Aldrich) was added (0.1 mg/mL), and the solution was incubated at 37°C for 6 hours. The digest was centrifuged at 12,000 *g*, at room temperature for 20 minutes, and the supernatant was precipitated with ammonium sulfate (60% saturation) and stored at 4°C. The precipitated material was collected by centrifugation (12,000 *g*, 4C, 30 min) and solubilized in 50 mM sodium phosphate buffer pH 6.0 containing 200 mM NaCl. The solution was dialyzed and concentrated with a 10-kDa molecular weight cutoff (MWCO) Amicon spin concentrator to a final volume of 5 mL. The solution was sequentially filtered through 0.45-micron and 0.22-micron filters before size exclusion chromatography on a HiLoad 16/600 Superdex column. Fractions containing BoNT/X were pooled and dialyzed against 50 mM sodium citrate buffer pH 5.0 and then applied to a cation exchange column (HiTrap SP HP (GE Healthcare:Cytiva)] and eluted with a sodium chloride gradient (0–1M). Fractions containing BoNT/X were dialyzed against 25 mM sodium phosphate buffer pH 7.4 and applied to a DEAE FF (Cytiva) column. Bound BoNT/X was eluted with a NaCl gradient (0–1M), and fractions containing the toxin were concentrated with an Amicon 100-kDa MWCO spin concentrator for analysis.

### Expression and purification of recombinant BoNT/X-A1 chimera

rBoNT/A (CAL82360.1) and rBoNT/X (BAQ12790.1) were synthesized as *E. coli* codon-optimized gblocks (IDT, San Diego, CA) consisting of the light chain and translocation domains (LC-HC_N_) of BoNT/X and binding domain (HC_C_) of BoNT/A1. The gBlocks were blunt ligated into pJET1.2 vectors (Thermo Fisher Scientific), PCR amplified via Phusion Hot Start Flex (New England Biolabs, Ipswich, MA) with overlap primers, assembled into a NdeI, XhoI linearized pET-15b vector in a three-fragment Hifi assembly (NEB) in fusion with the thrombin cleavable N-terminal his6 tag, and transformed into DH10B and then BL21 DE3 competent *E. coli* (New England Biolabs, Ipswich, MA). Whole plasmid sequencing via nanopore sequencing at ≥1,000× coverage was utilized to sequence verify the assembled construct.

To survey expression of the rBoNT/(X/A1)-pET-15b construct in Bl21, a 200-mL expression culture (LB + 100 µg/mL carbenicillin) was inoculated with overnight culture, grown at 37°C, 225 RPM, induced to 1 mM IPTG at OD600 0.4–0.6, and expressed for 24–27.5 hours at 16°C, 225 RPM. Whole cell lysate was evaluated for expression via SDS-PAGE Coomassie staining or western blot with 1° polyclonal α-A1 or α-X antibody or monoclonal α-His antibody (BioLegend 906102). Expression culture was pelleted at 6,000 × *g* for 8 minutes, resuspended in 50 mL sonication buffer [50 mM Tris, 50 mM NaCl, 20 mM imidazole, 5% glycerol, and protease inhibitor cocktail tablet (Pierce), pH 5.5], sonicated and loaded on to an equilibrated 2–3-mL Ni-IMAC column, washed to background with the same buffer, and eluted with 250 mM imidazole.

Peak NiNTA purified fractions of rBoNT/(X/A1) were trypsin treated at 1:50 trypsin at 37°C for 30 minutes. To acquire a rough minimum lethal dose for rBoNT/X/A1, NT/T-treated semi-purified rBoNT/X/A1 was serially diluted in 50 mM Tris, 50 mM NaCl at pH 5.5 at dilution factor 10 (1.0 μg to 100 pg). Dilutions were brought to 0.5 mL with gel phosphate as previously described and injected into 1 mouse per dilution. Mice were periodically monitored for up to 2 weeks post-injection.

### *In vitro* assays

#### hiPSC-derived cell lysate assay

hiPSC-derived motor neurons purchased from Fujifilm Cellular Dynamics (Madison, WI, USA) were prepared as recommended by the manufacturer. rBoNT/X holotoxin (isolated from *E. coli*) was pretreated with 10 mM DTT, then, toxin samples were treated with trypsin or left untreated, and serial dilutions were applied to hiPSC-derived neuronal cell lysate cultures for 1 hour. VAMP2 cleavage was analyzed by western blot using Synaptic Systems (Gottingen, Germany) Synaptobrevin2 antibody and goat anti-mouse Ig secondary (Santa Cruz).

To test for toxin neutralization by anti-BoNT/X antiserum, trypsin-treated rBoNT/X isolated from *E. coli* (20 nM) was incubated with anti-BoNT/X antiserum for 1 hour, and hiPSC-derived neurons were exposed to the antibody-BoNT/X mixture, BoNT/X alone, anti-BoNT/X antiserum alone, or culture media alone for 40 hours. Cell lysates were analyzed for VAMP2 cleavage via western blot, using syntaxin as a loading control.

#### Endopeptidase assay

Native BoNT/X derived from *C. botulinum* Strain 111 was assessed for *in vitro* cleavage of VAMP2. Human GST-VAMP2 2-94 was prepared as described previously (47). Briefly, cDNA from Hela cells was used as a template for the amplification of DNA encoding the full length of VAMP2. The amplified DNAs were inserted into pGEX6P3 vector (Cytiva). To delete the transmembrane region mutagenesis, PCR was performed. Recombinant proteins were expressed as C-terminally His-tagged and N-terminally GST-tagged proteins in *E. coli* strain BL21-CodonPlus (DE3)-RIL (Agilent Technologies) and purified using HisTrap HP (Cytiva) and GSTrap HP (Cytiva). BoNT/X was treated with reducing buffer (10 mM Tris-HCl pH 7.4, 20 mM NaCl, and 5 mM DTT) at 37°C for 30 minutes, before being diluted to estimated final concentrations of 1, 5, 10, 20, and 50 nM in cleavage buffer (10 mM Tris-HCl pH 7.4, 20 mM NaCl). BoNT/B1 was diluted to final concentrations of 5 and 50 nM. A control (0 nM) was treated as with all other samples but with no addition of toxin. Three-micromolar recombinant human GST-VAMP2 2-94 was added to each toxin dilution and incubated at 37°C for 45 minutes. Sample buffer was added, and samples were incubated at 95°C for 5 minutes before analysis via western blot. In follow-up experiment, reduced native BoNT/X was pre-treated at 37°C for 30 minutes with 100 mM EDTA, 1 μg mouse anti-BoNT/X antiserum, and 1 μg anti-BoNT/B rabbit polyclonal antibody or left untreated. Concentrations of BoNT/X (5 nM) and BoNT/B (100 nM) were selected to achieve approximately equal levels of VAMP2 cleavage. Toxins were diluted in reducing buffer (10 mM Tris-HCl pH 7.4, 20 mM NaCl, and 5 mM DTT).

### hiPSC-derived neuronal cell assays

hiPSC-derived motor neurons purchased from Fujifilm Cellular Dynamics (Madison, WI, USA) were prepared as recommended by the manufacturer. Neurons were exposed to serial dilutions of the isolated trypsin-treated or untreated rBoNT/X for 24 hours (for toxin expressed in *E. coli*) or 72 hours (for toxin expressed in *C. botulinum*) and analyzed for VAMP2 cleavage by western blot.

### Neuronal cell binding assay

Rat adrenal gland Phenochromocytoma (PC) 12 cells (provided by T. Kohda, Osaka Metropolitan University, Osaka, Japan) were seeded onto 3.5-cm dish coated with poly-L-lysine solution (Sigma-Aldrich) at a concentration of 3.0 × 10^5^ cells per dish. All culturing was performed at 37°C, 5% CO_2_. Cells were cultured 3 days (~70% confluent) before two successive PBS washes and addition of 0.5 mL ganglioside mixture (50 µg/mL in serum-free media) (Merck, Rahway, NJ). Cells were cultured for 24 hours and washed twice with PBS, followed by exposure to BoNT/X or BoNT/B (estimated 300 nM in 300 µL/well). After 4°C for 1 hour, the cells were washed three times with PBS to remove toxin. Samples were fixed with 4% paraformaldehyde at room temperature for 30 minutes, followed by three washes with PBS. Samples were permeabilized and blocked with 0.1% Triton- 1% bovine serum albumin (BSA, Sigma-Aldrich) at room temperature for 30 minutes. Add mouse polyclonal anti-BoNT/X antiserum and anti-BoNT/B rabbit polyclonal antibodies and incubate at room temperature for 1 hour, followed by three washes with PBS. Add Phalloidin-Alexa 568 (Thermo Fisher Scientific), Hoechst 33342 (DOJINDO, Kumamoto, Japan), and either anti-mouse secondary (for BoNT/X sample) or rabbit IgG-Alexa 488 (for BoNT/B sample). Repeat washing 3× with PBS, mount samples using anti-fade kit, and image with a CSUX1 Confocal scanner unit (Yokogawa, Tokyo, Japan) and IX71 Microscope (Olympus, Tokyo, Japan). The data were analyzed using Metamorph software (Molecular Devices, San Jose, CA).

### *In vivo* mouse assays

Unless otherwise noted, all ICR mice for rBoNT/X analysis were purchased from Envigo (Indianapolis, IN), while ICR mice for native BoNT/X analysis were purchased from SLC (Hamamatsu, Japan).

#### Mouse bioassay

The mouse lethality bioassay is the current gold standard for BoNT detection in the United States. Mice are injected i.p. with serial dilutions of toxin-containing samples and observed for symptoms indicative of neuromuscular distress including ruffled fur, labored breathing and wasp waist, muscle weakness and paralysis, or death (48–50).

rBoNT/X expressed 24 hours in *C. botulinum* Hall A hyper tox- culture was sonicated and solubilized, followed by either (i) collection of the extract supernatant (rBoNT/X supernatant) or (ii) purification of the extract via size exclusion followed by ion exchange chromatography and then pooling samples (rBoNT/X DEAE pool). Mice (*n* = 4) were injected intraperitoneally with 500 µL of trypsin-treated or untreated rBoNT/X supernatant (trypsin treatment 100 µg/mL, 30 minutes, 37°C) or rBoNT/X DEAE pool (trypsin treatment 10 µg/mL, 30 minutes, 37°C) and observed for 4 days.

Four-week-old female ICR mice (*n* = 3) (SLC) were injected i.p. with 5 or 50 µg native BoNT/X M-PTC in 300 µL 10 mM phosphate buffer (pH 6.0, 0.1% gelatin). Mice were observed for symptoms of motor-neuron impairment or other signs of distress at 6, 12, 24, 48, 72, 96, and 120 hours post-injection. As there were no mouse deaths associated with i.p. injections of recombinant or native BoNT/X, it was not possible to calculate a median lethal dose (LD50) for these experiments.

#### DAS Assay

DAS assays were performed essentially as per K.R. Aoki (34), wherein mice were injected in hindlimb gastrocnemius muscle using a 29 G needle (native BoNT/X; Terumo, Somerset NJ). The opposite hindlimb was injected with an equal volume of vehicle or 10 mM phosphate buffer pH 6.0, 0.1% gelatin (native BoNT/X). Abduction in the mouse hind digits was scored from DAS = 0 (normal) to DAS = 4 (maximal muscle weakness) at indicated timepoints.

## SUPPLEMENTAL MATERIAL

Supplemental Figures mbio.03106-23-s0001.docx Figures S1-S5.

ASM does not own the copyrights to Supplemental Material that may be linked to, or accessed through, an article. The authors have granted ASM a non-exclusive, world-wide license to publish the Supplemental Material files. Please contact the corresponding author directly for reuse.
