# Supplementary material for: Botulinum neurotoxin X lacks potency in mice and in human neurons
Source: mBio. 2024 Feb 13;15(3):e03106-23. doi: 10.1128/mbio.03106-23 (PMC10936432; doi:10.1128/mbio.03106-23)
Supplement: Supplemental Figures — Figures S1-S5. [file mbio.03106-23-s0001.docx]

## SUPPLEMENTAL MATERIALS:


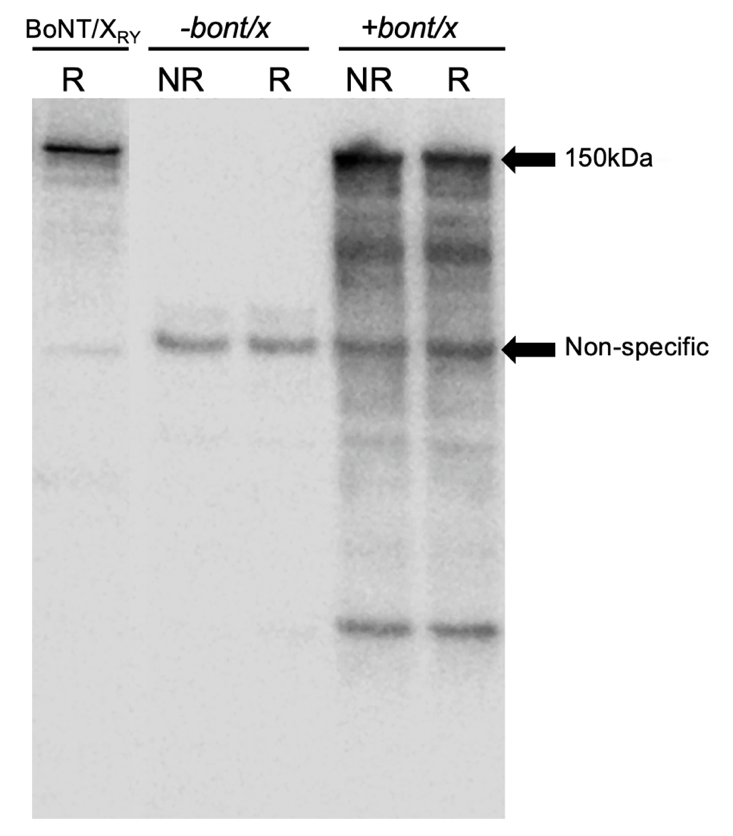


**Supplemental Figure 1:** Mouse anti-BoNT/X antiserum detects recombinant BoNT/X produced in *C. botulinum*. Wild type recombinant BoNT/X was expressed in atoxic *C. botulinum* Hall A Hyper tox ^-^, and total culture of the host strain without (-*bont/x*) or with (+*bont/x*) the *bont/x* containing plasmid was examined by Western blot using anti-BoNT/X antiserum produced in mice. 60ng BoNT/X_RY_ produced in *E.coli* was used as a control. The antiserum recognized BoNT/X expressed in *C. botulinum*, and only one non-specific band in the expression host not expressing BoNT/X was detected.

R: reduced, NR: non-reduced


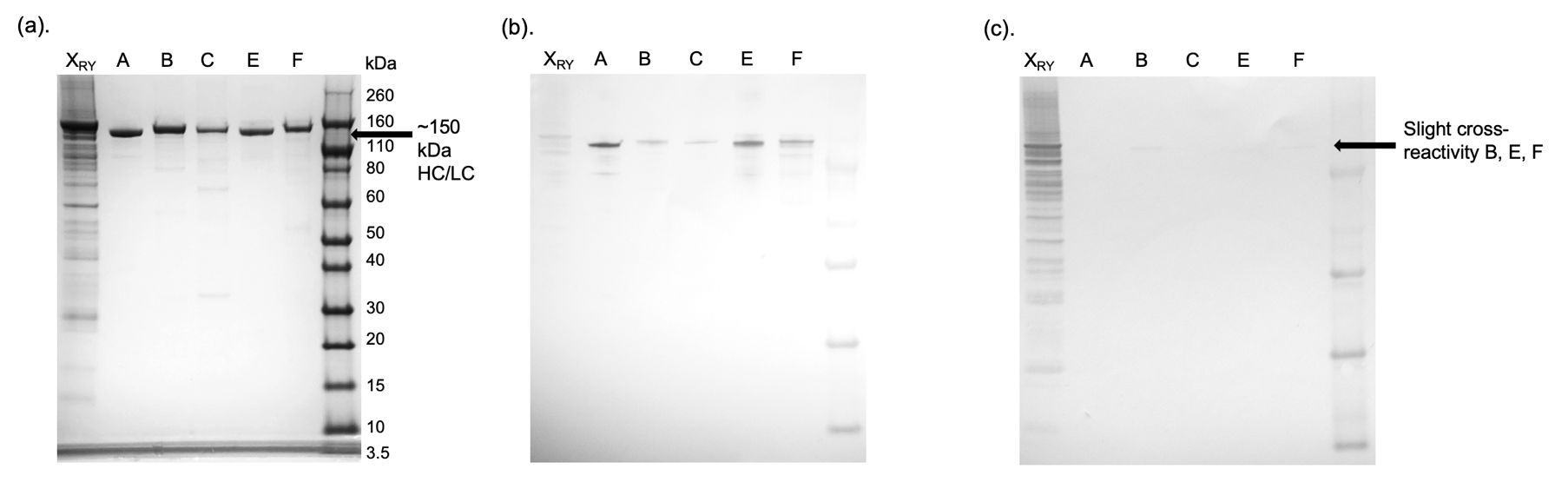
**Supplemental Figure 2: Cross-reactivity of BoNT/X with anti-BoNT/A/B/C/E/F antibodies**. ~1 $\mu g$ toxin of serotypes A,B,C,E,F, and ~ 5 $\mu g$ of catalytically inactive BoNT/X toxin (X_RY_) were analyzed via SDS-PAGE (a). Western blot analysis of 50 ng each toxin probed by a cocktail of 0.5 $\mu g /mL$ each of rabbit polyclonal anti-type A,B,C,E,F antibodies (protein A purified) (b) or anti-BoNT/X antiserum (c) and developed with BCIP. Types B, E, and F toxin reacted minimally with anti-BoNT/X antiserum. There was no observed reactivity with serotypes A or C.


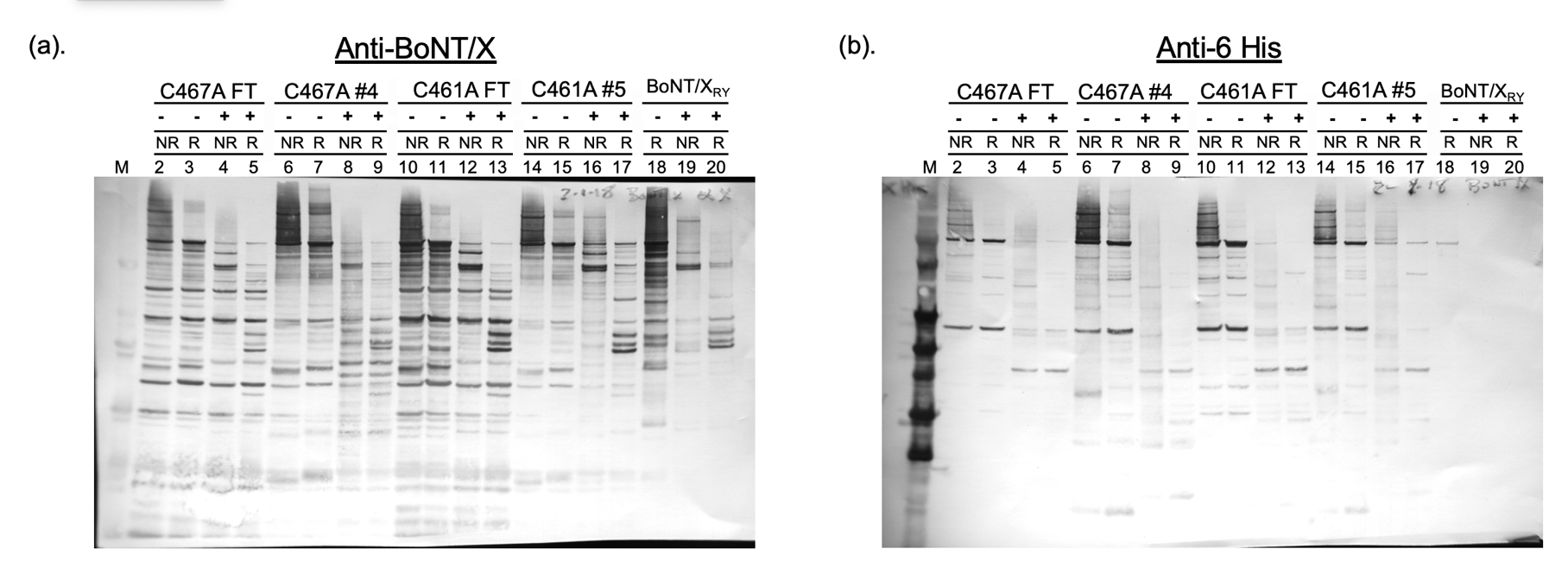


**Supplemental Figure 3: Mutation of C467A and C461A in BoNT/X_RY_ did not improve aggregation in un-reduced samples.** Two point mutations were made to C467 and C461, and samples were assayed via Western blot probed with anti-BoNT/X antiserum **(a)** or anti-6 His antibody **(b).** There was no observed difference detected in the Western blot patterns of the cysteine mutants or BoNT/X_RY_, and both showed aggregation in non-reduced samples that resolved upon reduction with DTT. Additionally, there was no observed difference in trypsin cleavage patterns between cysteine mutated BoNT/X_RY_ and BoNT/X_RY_.

R: reduced, NR: non-reduced, +: trypsin treated, -: non-trypsin treated, M: Novex sharp marker, 2/3:C467A IMAC flow through -/+DTT, 4/5:C467A IMAC flow through + Trypsin -/+DTT, 6/7:C467A IMAC bound frac 4 -/+DTT, 8/9:C467A IMAC flow through bound fraction 4 + Trypsin -/+DTT, 10/11:C461A IMAC flow through -/+DTT, 12/13:C461A IMAC flow through + trypsin -/+DTT, 14/15:C461A IMAC flow fraction 5 -/+DTT, 16/17:C461A IMAC flow fraction 5 +Trypsin -/+DTT, Control BoNT/X_RY_

**
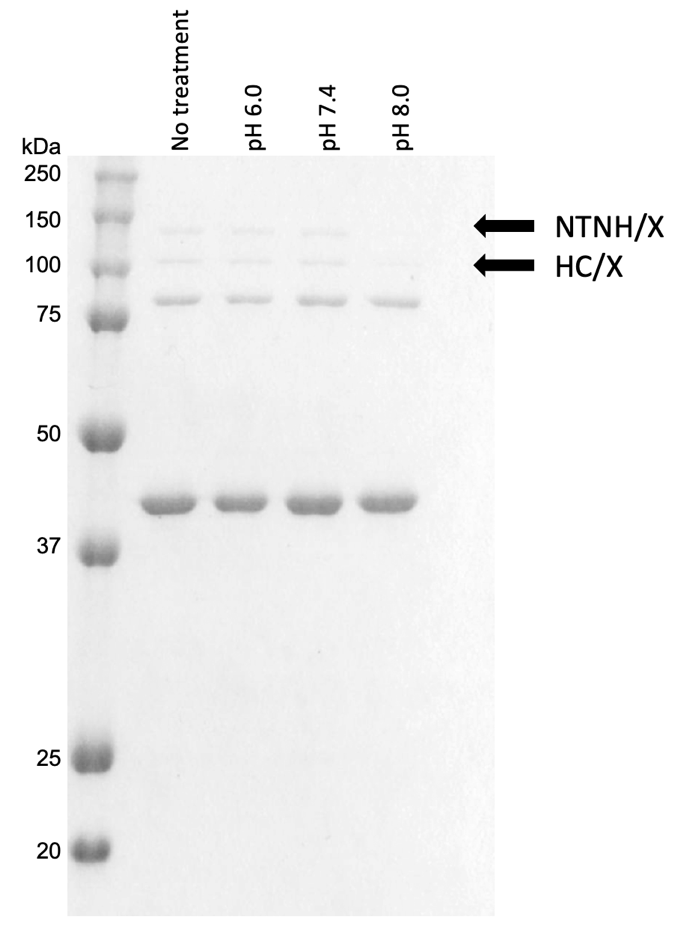
**

**Supplemental Figure 4: BoNT/X dissociated from minimal progenitor complex (M-PTC) is unstable at pH 8.** Native BoNT/X M-PTC incubated 10 hours at room temperature in 50 mM phosphate buffer of pH 6.0, 7.4, or 8.0. Upon treatment with pH 8.0, BoNT/X was unstable

**
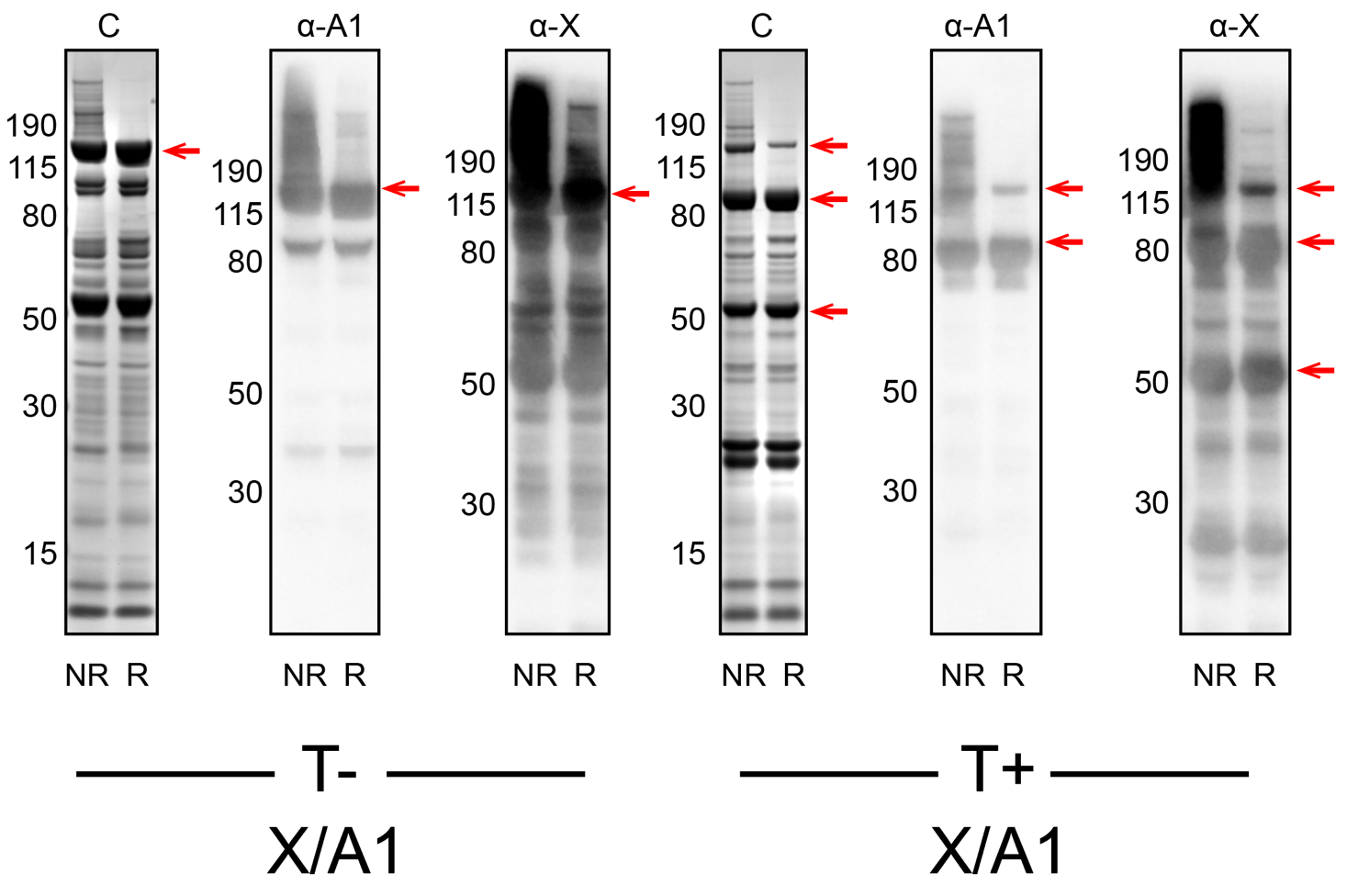
**

**Supplemental Figure 5:** Recombinant X (LC-HCN)/A(HCC) (X/A1) chimera was expressed in *E. coli*, and purification products analyzed via SDS-PAGE gel stained with Coomassie (C). Western blots were probed with either anti-BoNT/A1 polyclonal antibody (𝞪 A1) or anti BoNT/X polyclonal antibody (𝞪 X). Samples were treated with trypsin (T+) or left untreated (T-) , and unreduced (NR) or reduced via DTT (R). Arrows indicate ~150kDa holotoxin, ~100 Heavy chain, and ~50 kDa Light chain. Densitometry of the Coomassie-stained gel indicated ~20% purity of the BoNT/(X/A1).
